# A Knowledge-Based Cognitive Architecture Supported by Machine Learning Algorithms for Interpretable Monitoring of Large-Scale Satellite Networks

**DOI:** 10.3390/s21134267

**Published:** 2021-06-22

**Authors:** John Oyekan, Windo Hutabarat, Christopher Turner, Ashutosh Tiwari, Hongmei He, Raymon Gompelman

**Affiliations:** 1 Department of Automatic Control and Systems Engineering, University of Sheffield, Portobello Street, Sheffield S1 3JD, UK; j.oyekan@sheffield.ac.uk (J.O.); w.hutabarat@sheffield.ac.uk (W.H.); a.tiwari@sheffield.ac.uk (A.T.); 2 Surrey Business School, University of Surrey, Guildford, Surrey GU2 7XH, UK; 3 School of Computer Science and Informatics, De Montfort University, The Gateway, Leicester LE1 9BH, UK; mary.he@dmu.ac.uk; 4 IDirect UK Ltd., Derwent House, University Way, Cranfield, Bedfordshire MK43 0AZ, UK; rgompelman@idirect.net

**Keywords:** satellite networks, machine learning, IoT

## Abstract

Cyber–physical systems such as satellite telecommunications networks generate vast amounts of data and currently, very crude data processing is used to extract salient information. Only a small subset of data is used reactively by operators for troubleshooting and finding problems. Sometimes, problematic events in the network may go undetected for weeks before they are reported. This becomes even more challenging as the size of the network grows due to the continuous proliferation of Internet of Things type devices. To overcome these challenges, this research proposes a knowledge-based cognitive architecture supported by machine learning algorithms for monitoring satellite network traffic. The architecture is capable of supporting and augmenting infrastructure engineers in finding and understanding the causes of faults in network through the fusion of the results of machine learning models and rules derived from human domain experience. The system is characterised by (1) the flexibility to add new or extend existing machine learning algorithms to meet the user needs, (2) an enhanced pattern recognition and prediction through the support of machine learning algorithms and the expert knowledge on satellite infrastructure, (3) the ability to adapt to changing conditions of the satellite network, and (4) the ability to augment satellite engineers through interpretable results. An industrial real-life satellite case study is provided to demonstrate how the architecture could be used. A single blind experimental methodology was used to validate the results generated by our approach.

## 1. Introduction

There has been an increase in the number of satellites launched into space in recent years. For example, recently, SpaceX launched 60 internet-beaming satellites [1]. This will bring great advantages to global positioning for navigation, remote sensing, and telecommunication between remote points on Earth, among other uses, as well as increasing global connectivity and further enabling applications that rely on Internet of Things technology. Maritime transportation is one of the main application domains of satellite communication. Over 90% of worldwide trade is served by the maritime market [2]. Vessels at sea regularly utilise satellite communications to stay in touch with operations on land, and about 47.15% of the vessels are out of coverage of the coastal AIS transceiver and would rely on the connectivity to a satellite system [3]. Satellite services offer the advantages of increased productivity and improvement in the quality of work for over one million seafarers, over 20,000 vessels are currently online through a broadband satellite communications connection, and over 50,000 vessels are expected to be connected by 2025 [4]. At over EUR 2.5 billion in retail revenue in 2018, growing at an average 7.8% over the next ten years, the seas have the potential to generate significant connectivity revenues for the satellite industry [5].

Currently, network monitoring is still heavily dependent on humans using a reactive approach (that is, when a customer calls to report a fault). Engineers use their expertise to decide what attributes and values provide or predict a snapshot of a satellite infrastructure’s health. For example, if the attribute is outside the specified range it might indicate poor terminal condition, e.g., faulty or about to develop a fault.

However, with the proliferation of IoT devices, the number of terminals is set to increase, and this approach quickly becomes unmanageable by human operator alone. A single threshold value indicating terminal health does not fit all scenarios as each terminal in a network has unique operating conditions. Moreover, generating a threshold for each terminal separately is not feasible for more than a million terminals in the satellite network [4].

In this work, a knowledge-based cognitive architecture is proposed that can autonomously monitor a satellite infrastructure with a large number of terminals towards achieving two objectives:

(i) Predictive maintenance is to keep track of a terminal’s degrading signal quality to timely verify equipment in order to anticipate eventual problems that may lead to higher costs with corrective maintenance. Hence, it could reduce the maintenance cost, device failures, downtime for repairs, and human workload.
(ii) Reactive maintenance is to automatically inform network operators when a terminal suddenly drops out of operation, e.g., due to a sudden hardware failure, so that engineers can attempt onsite repairs quickly before a customer calls. Hence, it could greatly reduce the unexpected consequences brought to the customers, thus maintaining a high quality of services and improving satellite network operator and customer relations. In the context of this work, the factors that could lead to abnormality of satellite networks are:
  (1) Environmental factors—obstacles around satellite dishes, cloud coverage, and ageing subcomponent hardware issues. High cloud coverage has usually temporary effects, although there are a limited set of actions that engineers can use to mitigate the effects. Moreover, obstacles around the dishes such as trees might cause intermittent drops of signal quality caused by branch waving. Such cases, although temporary, might have a high impact on connectivity and be difficult to detect.
  (2) Device health factors—ageing subcomponent hardware issues could also cause a long-term degradation of a terminal’s performance over time. Identifying and detecting all the events discussed above in a satellite network with more than a million nodes is beyond the capability of a human. Hence, an automated system is required to release the human’s cognitive load.

In order to understand network status and predict potential situations that might disrupt a network, an Endsley situational awareness model, proposed by Sotelo et al. [6], was applied as a starting point for a knowledge-based cognitive system. In the Endsley situational awareness model, the role of a domain knowledge base in the cognitive process is missing.

In this paper, a knowledge-based cognitive architecture is proposed that merges expert knowledge on the satellite infrastructure with the ability of machine learning algorithms to detect patterns from large amounts of data. The architecture is able to detect possible anomalies in the satellite infrastructure. The implemented architecture has (i) flexibility that enables to extend or replace existing machine learning algorithms to match the needs of the user; (ii) the ability to use knowledge of the satellite infrastructure to guide the machine learning algorithms during training and online deployment of the trained models; (iii) the capacity to adapt to the changing health conditions of a satellite network, and finally, (iv) the capacity to provide interpretable results to infrastructure engineers.

The remainder of this paper is organised as follows. In Section 2, the related work is surveyed. In Section 3, a knowledge-based cognitive architecture is presented; this is followed by results of single-blind experiments carried out on an industrial case study using anonymised real satellite infrastructure data in Section 4. Further, an overview discussion is provided in Section 5, and, finally, the conclusions are given in Section 6.

## 2. Related Work

Cyber–physical systems such as satellite networks generate a vast amount of temporal traffic data, which may carry crucial information on when and why faults usually occur [7]. However, proactive fault detection in the satellite communication industry is still in its infancy. Furthermore, providing real-time anomaly detection in satellite networks has not been achieved according to the authors’ knowledge.

This is because, first, the data generated in satellite networks are dynamic and multivariate. The temporal aspect of the data and continuously changing operating conditions of a satellite network makes a machine learning training process difficult because patterns recognised a few months ago might be rendered obsolete. Moreover, event detection in time series data is often complicated by the inherent nature of the data sets in that they exhibit the potential for multiple patterns to be discovered from the interactions between individual variables and may not be traceable far back within a data set [8].

Second, a lot of robust processes are followed during the design and development of a satellite. This is due to the fact that once launched, it is difficult to effect repairs on a satellite. As a result, a satellite network operates normally without faults for most of the time. The rare occurrence of faults makes it challenging to train supervised machine learning algorithms (algorithms that require human labelled data). Additionally, it is likely that the architecture will experience events that were not previously thought of during the satellite design and development process.

**Figure 1:**
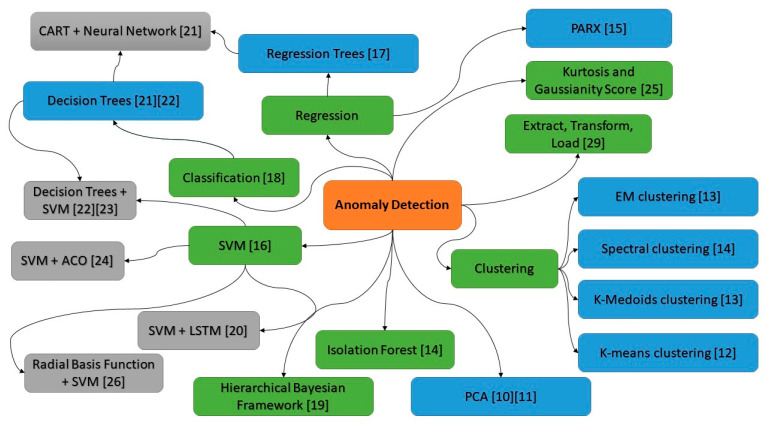
Mind map of the concepts from the literature review used for anomaly detection [10,11,12,13,14,15,16,17,18,19,20,21,22,23,24,25,26,27,28,29].

There has been research in other domains that could potentially be applied to the challenges discussed above. For example, the dynamic and multivariate aspect of data can be addressed by applying dimensionality reduction techniques. A classic approach for linear dimensionality reduction in data sets is the principal component analysis (PCA) [9] (Figure 1). PCA is a statistical procedure that uses an orthogonal transformation to convert a set of observations of possibly correlated variables into a set of values of linearly uncorrelated variables. This enables a user to focus on a handful of domain independent variables that have a high impact on the dependent variable being investigated. As a result, PCA is usually the first stage in anomaly detection. For example, Kudo et al. [10] proposed a PCA-based robust anomaly detection scheme by using the daily or weekly periodicity in traffic volume. In the proposed scheme, traffic anomalies are detected for every period of measured traffic via PCA in the Abilene network, where the outliers in the current period are removed by means of a reference covariance matrix from normal traffic in the preceding period; To avoid the high requirements of computational cost and memory for storing the entire data matrix or covariance in classic PCA, Bagane and Patil [11] proposed an oversampling PCA to reduce dimension of data for anomaly detection through duplicating the target instance multiple times to amplify the effect of outliers.

Due to unknown or unforeseen cases and the challenge of labelling a large amount of data, unsupervised learning techniques (techniques that do not require human labelled data), e.g., clustering-based approaches, have been developed for anomaly detection. For example, Munz et al. [12] deployed the k-mean clustering algorithm in order to separate time intervals with normal and anomalous traffic in the training dataset. The resulting cluster centroids were then used for fast anomaly detection in monitoring new data. Syarif et al. [13] showed that an EM clustering algorithm for anomaly detection achieved a better performance than the k-means and k-medoids clustering algorithms. The EM clustering algorithm assigns an object to a cluster according to a weight representing the probability of membership. In addition, Habeeb et al. [14] developed a streaming sliding window local outlier factor coreset clustering algorithm, and obtained better performance than existing algorithms such as k-means, isolation forest, spectral clustering, and agglomerative clustering.

The problem of anomaly detection can be modelled as a regression problem or classification problem. Regression is the process of estimating the relationships between a dependent variable and one or more domain independent variables. The use of regression enables users to use models to predict into the future on what pathways the variables of a system might be going and what effect the result will have on the system. For example, Liu and Nielsen [15] proposed regression-based online anomaly detection for smart grid data by combining a short-term energy consumption PARX (prediction algorithm, periodic autoregression with exogenous variables) algorithm and Gaussian statistical distribution. The study of Kromanis and Kripakaran [16] showed that support vector regression models that predict structural response from distributed temperature measurements could form the basis for a reliable anomaly detection methodology. Khoshgoftaar et al. [17] successfully deployed nonlinear regression trees for fault finding in software systems by predicting which software modules are most likely to be faulty during the development phase of the software lifecycle.

Anomaly detection can also be modelled as a classification problem, which is the process of assigning a category or a “class” to the new observation. Recently, classification-based methods were shown to achieve superior results on anomaly detection. Bergman and Hoshen [18] proposed a novel method that unifies the current classification-based approaches to overcome generalisation issues for nonimage data by extending the transformation functions to include random affine transformations. Roberts et al. [19] provided a unified statistical approach to classification and anomaly detection within a hierarchical Bayesian framework, and improved the performance of dealing with uncertainties by marginalising over the unknown true value of the data.

Hybrid approaches are often used for anomaly detection. For example, Al-Mamuna and Valimaki [20] proposed a two-stage approach to anomaly detection for quality control in cellular networks. The first stage was to create a one-class SVM model to find outliers in the dataset of key performance indicators (KPIs) from all the cells (sectors of each 2G/3G/4G/5G base station). The second stage was to use a long short term memory (LSTM) recurrent neural network for further understanding of their behavior. Capozzoli et al. [21] deployed fault detection model CART (classification and regression tree) joined with a neural network. Samantaray [22] deployed a decision tree and SVM to identify faults in an electricity transmission infrastructure. It is believed that decision trees have the characteristics of a transparent decision-making process, making results interpretable. Moeyersoms et al. [23] utilised random forests and SVM for fault diagnosis in software development projects, and visualise decision trees to explain how the decision model came to its conclusions.

SVMs have been used with varying degrees of success for detecting the status of networked systems. Feng et al. [24] developed an algorithm that combined SVM with ant colony optimisation to protect networked systems from attacks in real time. Interestingly, the authors noted that the improvement in intrusion detection was only observed when algorithms were used together. Applying SVM only resulted in lower average detection rate, and higher false negative and false positive rates. Similarly, applying only ant colony optimisation required longer training time and resulted in comparable detection rates and false alarm rates. Purarjomandlangrudi et al. [25] developed an anomaly detection algorithm based on kurtosis and non-Gaussianity score, which achieved better accuracy than SVM when detecting rolling-element bearing failures. The reason why SVM algorithms do not perform well when deployed alone is their reliance on labelled data. As mentioned earlier, anomalies usually do not occur frequently, resulting in difficulty in learning fault patterns. Moreover, the training data are unlikely to cover all potential faults that might occur during operations. In SVMs, knowledge is embedded as kernels, which can be represented in a variety of forms, and their parameters must be tuned accurately in order to represent the domain. However, some of the parameters are sensitive and make the models inaccurate, hence, directly affecting the accuracy of decision making. For example, a radial basis function (RBF) kernel was proposed by He et al. [26] in an incremental SVM based on information gain to find the best features for spam detection.

The ability to accurately predict future fault states and behaviors from current temporal events in (near to) real time could avoid severe consequence from potential incident events. However, performing analysis in quasi-real time (that is a close approximation to actual real time) but is one of the major challenges in mining time series data Gaber et al. [27]. This challenge was addressed by Weiss [28] using a basic error detection computational agent to mine telecommunication data for network faults. However, as there was a lack of embedded knowledge of networks in their approach, the high frequent alerts for both potential problems (false positives) and real problems made it hard to analyse by human means alone. In practice, the cognitive (information processing) load on the human was not reduced. The human was overwhelmed and not “augmented or served” by the system.

One of the possible solutions to address changing operational conditions and low frequency of faults is to incorporate the domain knowledge in the pattern detection process. For example, Kalegele et al. [29] proposed utilisation of the domain knowledge of network management software systems by ETL (extract, transform, load) units during data mining. This enabled the unit to improve the value of the insight generated from system operational data. The embedding of domain knowledge in computational agents has the following advantages: (a) it is in a form that a human can comprehend, (b) it is capable of representing the domain accurately, and (c) the knowledge model’s parameters are not sensitive to outliers.

From the above, it can be seen that there are various techniques that could be applied to detecting anomalies in satellite networks. Each approach has its strengths and weaknesses, and one technique cannot fit all; moreover, domain knowledge is important for anomaly detection to improve the usability of the system. There is a need to have a system that can accommodate multiple techniques and domain knowledge to deal with various issues in the real world.

Currently, a typical human engineer, when looking for anomalies in the network, uses a combination of techniques and domain knowledge in troubleshooting a satellite network. As a cognitive agent, the human builds his or her expertise over time in the form of a knowledge base that can be applied to novel faults. Following this line of thought, we propose a knowledge-based cognitive architecture capable of embedding multiple algorithms and addressing the limitations highlighted above.

## 3. The Proposed Architecture

### 3.1. The Satellite Network Infrastructure

**Figure 2:**
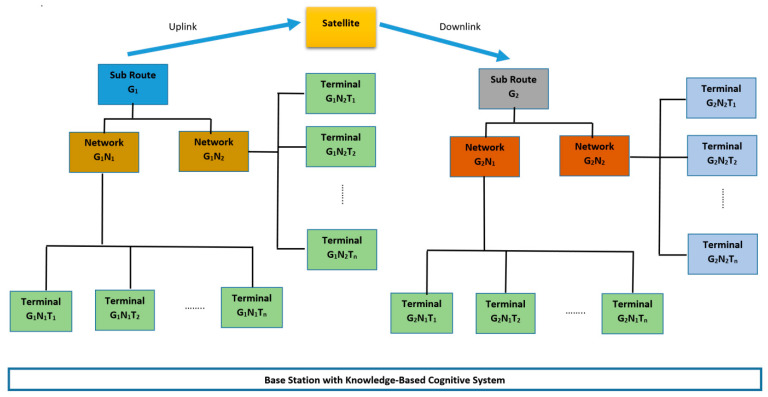
The hierarchical architecture of satellite networks.

In order to develop an effective knowledge-based cognitive architecture, we start with understanding the domain in which the architecture will be operating- a satellite network infrastructure. The satellite network infrastructure for this study is made up of sub route groups, $G_{i}\in \left\{G_{1},\;G_{2},\;G_{3}\ldots \ldots .G_{N}\right\}$, and each sub route group *Gi*, is linked to several networks $G_{i}N_{k}\in \left\{G_{i}N_{1},\;G_{i}N_{2},\;G_{i}N_{3}\ldots \ldots .G_{i}N_{N}\right\}$, where *Nj* is a network of terminals, represented as $G_{i}N_{k}T_{j}\in \left\{G_{i}N_{k}T_{1},\;G_{i}N_{k}T_{2},\;G_{i}N_{k}T_{3}\ldots \ldots .G_{i}N_{k}T_{N}\right\}$, as shown in Figure 2.

Together with the application data packet to be sent, each terminal, $G_{i}N_{k}T_{j}$, sends status data packets with a set of attributes, such as id, downstream signal to noise ratio, upstream signal to noise ratio, temperature of the terminal, latitude and longitude, etc. As shown in Figure 2, the status data packets are received at a base station for network monitoring purposes. Each sub route group, $G_{i}N_{k}$, transmits high level statistics data with a larger set of attributes, including the attributes of the sub route group, such as id, maximum transmit power of the group, IP-address, customer id, transmit carrier frequency, receiving carrier frequency, and satellite name, among other variables. Each terminal, $G_{i}N_{k}T_{j}$, in a sub route group, $G_{i}N_{k}$, can transmit and receive data. Figure 2 illustrates the hierarchical architecture of the satellite network.

### 3.2. The Framework of the Proposed Architecture

In order to understand network status and predict potential situations that might disrupt a network, an Endsley situational awareness model, proposed by Sotelo et al. [6], was applied as a starting point for a knowledge-based cognitive system. In the Endsley situational awareness model, the role of a domain knowledge base in various modules of the cognitive process is missing.

**Figure 3:**
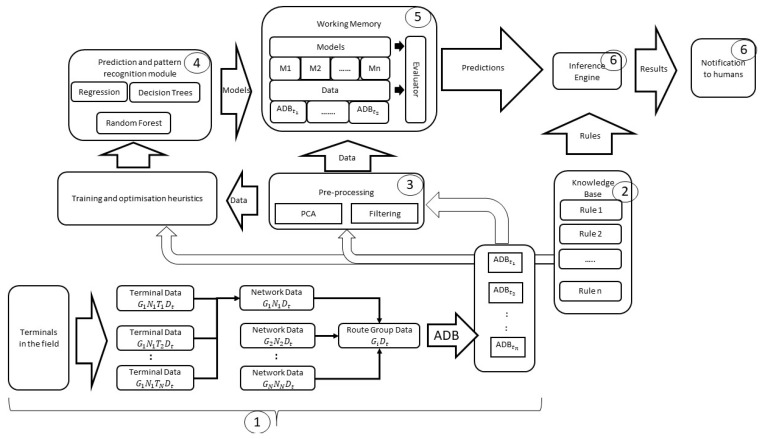
Proposed framework of the architecture.

Figure 3 illustrates the framework of the proposed architecture. The arrow from the knowledge base module to the training algorithms implies that rules from the knowledge base are fed to the training algorithms to produce the heuristic strategies that are used to reduce the speed of training. Using the knowledge base rules in the pre-processing component enables us to improve its performance and sense-check the results of this module. The numbers in bubbles correspond to sub-section numbers in Section 3.3 (The Modules in the Knowledge-Based Cognitive Architecture).

In our work, a knowledge-based cognitive architecture is proposed by applying the knowledge base to various stages of the cognitive process in the Endsley situational awareness model. The proposed architecture includes five functional modules: (1) a flexible prediction and pattern recognition module that enables addition and extension of existing machine learning techniques for anomaly detection—this supports the ability to configure the architecture based on user needs and support offline algorithm training process; (2) a knowledge base module made up of rules derived from the expert’s domain knowledge of satellite networks; (3) the data pre-processing module, which has the capacity to remove noise, perform feature extraction or dimension reduction, etc.; (4) the working memory module which is used for storing the data collected from the networks to be monitored and models produced by the machine learning algorithms; and (5) an inference engine with an interface that produces (visual) outputs and allows user involvement in the decision making.

### 3.3. The Modules in the Knowledge-Based Cognitive Architecture

We shall now discuss the modules in the architecture presented in Figure 3, with the numbers corresponding to the module number in the architecture:

**The monitored network** (1): In this work, satellite terminals were mostly static and situated in various geographical locations worldwide. Each terminal transmitted data in intervals of 0.3 s. The data received at base stations consisted of the aggregated data bundle (ADBt) from all sub route groups. Each aggregated data bundle comprised terminal data ($G_{i}N_{k}T_{j}D_{t}$), which were produced every 0.3 s, where t is the timestamp (see Figure 2).

**Knowledge base** (2): The knowledge base was made up of rules derived from a team of 10 satellite engineers with a combined experience of more than 80 years. These rules were derived based on: (i) the knowledge of the satellite infrastructure (Figure 2), (ii) experience on the behaviour of terminals in networks, (iii) experience of signal propagation in satellite networks, and (iv) the behaviour of terminals at various geographical locations.

Deriving rules from knowledge of satellite infrastructure and behaviour of terminals in networks (i and ii): One of the important rules applied in the knowledge base was closely linked to the satellite infrastructure design (Figure 2). From experience, satellite engineers discovered that terminals in the same network exhibited similar behaviour. As a result, when a terminal in a network was not behaving the same as the other terminals in the network or exhibited behaviour similar to another network, this was a cause for concern. However, capturing the behaviour of networks was a task that was not humanly possible using simple if–else rules. This is because a network $G_{i}N_{k}$ is made up of various terminals $G_{i}N_{k}T_{j}$ with different hardware behaviours (e.g., age of equipment, different component manufacturers, etc.) and unique geographical location conditions (e.g., weather conditions, trees growing close to terminals could obstruct line of sight to satellite, etc.) that is not common across all terminals. As a result, we had to use a variety of machine learning algorithms to model network behaviours and use the models to support the derivation of the following rule: if a terminal is predicted to belong to another network different from its assigned network, then it is assumed that either the model that did the prediction is wrong or there is something wrong with the terminal.

Deriving rules from experience of signal propagation in networks and behaviour of terminals at geographical locations (iii and iv): From experience, the satellite engineers were able to identify three different states that a terminal in the network could be in—a normal working state, a transient state with cloud covering or tree waving in front of satellite dish (i.e., geographical conditions), and an abnormal (broken down) state. In order to detect the state of a terminal, the engineers had previously identified a signal propagation variable and used it as a threshold value in an if–else rule structure to assess the state of a terminal. Furthermore, by using the value of this variable, it was possible to assess and estimate certain short transient conditions (passing cloud covering or rain), slow but continuous spatiotemporal conditions (tree growth) happening in the vicinity of the terminals, or a total hardware failure caused by a broken terminal. However, this approach caused a multiple of problems. For example, due to the differences in hardware of the terminals, one threshold value could not be rolled out to over 1000 terminals in the network. This called for an automated process of extracting this threshold value for each terminal in the network. Moreover, it was discovered that through the use of machine learning algorithms, other signal propagation variables were discovered. It was discovered that these variables could be used in combination with the variable that the engineers were using previously.

**The pre-processing module** (3): In this module, the data from the satellite network are retrieved and pre-processed. One of important pre-processing steps is data filtering. It refers to the process of defining, detecting, and correcting errors in raw data, in order to minimise the impact on subsequent analyses. There may be multiple filters. The techniques used for filtering depend on the requirements of data analysis and the data itself. In this work, the filters applied in this module focused on removing missing attribute values caused when a sensor broke down in a terminal. After this initial filtering, PCA was applied for dimensionality reduction. The results of the dimension reduction were then sense-checked using rules from the knowledge base that embeds domain specific knowledge. For example, as mentioned before, it was reveal through PCA that a set of signal propagation variables could be used in combination to predict the state of a terminal.

**The prediction and pattern recognition module** (4): As noted in the literature review, many machine learning techniques can be used for anomaly detection. Nevertheless, no matter what kind of technique is used, function mapping is needed to represent the relation between the output y and the input features ($x_{1}$, …$\;x_{n}$), which are the observed variables in the network (Equation (1)). This function can be represented with any type of machine learning model. In this work, a variety of machine learning algorithms were applied. These included supervised learning techniques such as decision trees and support vector machines, and unsupervised learning techniques such as k-means and k-nearest neighbour.

*y = f(x1,…xn)* (1)

The prediction and pattern recognition module provided us the opportunity to load and test various algorithms for tests. Algorithms for pattern recognition enabled us to find regular patterns in data. Through these regularities in the data, models of the patterns seen in the domain can be built and used to classify terminal behaviour based on the value of their attributes. Examples of such algorithms used in this work include decision trees and k-means. The derived models of the domain were then used to predict which class (disrupted or not disrupted) a terminal might fall into depending on the value of its attributes.

The prediction algorithms used in this work attempted to fit curves to data during training. These curves are mostly mathematical models that have a dependent target that rely on the values of attributes of the terminals. During training, the prediction algorithms work by using a performance measure (cost function) to minimise the error of the curve in relation to the modelled dataset. The output of the training is a mathematical model that can then be used to either extrapolate or interpolate for those data points that are not present in the data set. Examples of prediction algorithms used in this work are linear regression and SVM regression. The advantage of using the framework shown in Figure 3 is that it gives the capability to compare different algorithm performances for further application.

Both classes of algorithms noted above were used to build prediction and pattern recognition models. In this work, the algorithms that produced the best models were carried forward. In determining which model to carry forward, the working memory was used.

**Working memory** (5): In our architecture, the working memory was used to store models generated by the machine learning algorithms and aggregated data bundles (ADBts) derived from the monitored network. The data were saved in a way of first-in–first-out (FIFO). The data were used by the architecture to generate models via its prediction and pattern recognition algorithms. The models generated were assessed by the evaluator in the working memory. Various performance criteria (comprising accuracy, precision, and recall values) were used to evaluate each model and determine which model to take forward. Furthermore, the performance criteria enabled tests on the generated models using real-time data to assess if they need to be recreated to take new data into account. Because these models could go “stale” over a period of time, another criterion which will be tested in future work is using a time threshold above which the models will need to be regenerated using the latest data.

**Inference engine with user interface** (6): The inference engine provided a sense-check of the predicted results from the generated models in working memory. The rules present in the knowledge base were used in making various checks. By using these rules, it was possible to infer the status of terminals as well as derive an understanding of the conclusion reached by the architecture. An interface provides users with a chance to visualise and interact with the results of the architecture. This enables users to be involved in the final decision using the architecture’s results.

### 3.4. Architecture Implementation

**Figure 4:**
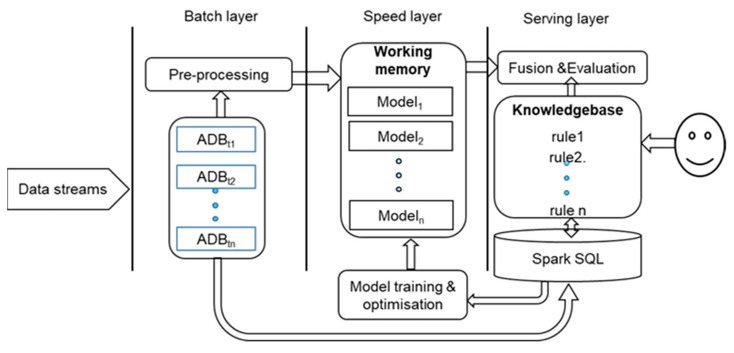
The system implementation under the lambda architecture.

The proposed knowledge-based cognitive architecture was implemented using Amazon Web Services (AWS). AWS provides many of the building blocks required to build a secure, flexible, cost-effective lambda architecture in the cloud. A lambda architecture describes a system consisting of three layers: (1) a batch layer runs a batch job iteratively, and data arriving after a job starts is processed by the next job; (2) a speed layer directly retrieves data streams from data sources, processes them, and updates the results into the real-time views in the database in the serving layer, and it does not keep any history records, and typically uses main memory-based technologies to analyse the incoming data; and (3) a serving layer is to provide responses to users’ queries. Hence, in the serving layer, users can be allowed to input their points of view in terms of the views from the speed and the batch layers, and the system fuses them to output final decisions. Therefore, we can create our proposed architecture by combining AWS services with Apache Spark Streaming and Spark SQL for anomaly detection based on live data streams. Figure 4 illustrates the implementation our knowledge-based cognitive architecture under the lambda architecture. In the batch layer, the batched data were pre-processed, the pre-processed data (features) are input to the trained models in the speed layer, and the outputs of the models are sensed-checked with the rules in the knowledge base. Finally, the results are shown to users for evaluation.

## 4. Case Study

An industrial case study was used to demonstrate the effectiveness of the architecture and validate it. Data were provided by the industrial partner on this project. However, the variables of the dataset were coded in order to guard against issues with intellectual property. However, we provided some indication of what the variables represent in the name of the code we used to code the variables of the dataset. Furthermore, a single-blind study was applied. The data scientists and architecture implementers did not know and were deliberately kept unaware of where and when disruptions occurred at the terminal, network, or sub route group level. Furthermore, they did not have access to the data on the geographical conditions (tree growths, cloud cover, short-term transients) at the sites where the terminals were located. However, the satellite engineers had access to this knowledge. This ensured that the result produced by our architecture was not biased. Only after experiments were conducted and results produced by the data scientists and the architecture implementers were the satellite engineers able to reveal and confirm the results. This approach provided validation of the results produced in this work.

### 4.1. Obtaining Data from the Networks

Three datasets containing data for sub route groups, networks, and terminals were used in the experiments. The data set was divided into 20 file batches, each containing over 100K rows of data timestamped events. At this stage of the research, training was performed offline and attempts were made to find anomalies in the dataset (in future work, real-time deployment in a production environment will be implemented). Due to confidentiality, the variables used in this work were anonymised. The sub route groups are called SUB_GRP_X and network NET_X (Table 1).

### 4.2. The Pre-Processing

Using the domain knowledge of the satellite network, a filtering algorithm was developed to filter data rows in the dataset that contain spurious values. For instance, this included null values caused by the terminal breaking down, and some timestamped events that did not record the terminal ID were filtered out by the pre-processing module in our architecture. It is noted that data samples that omitted terminal ID accounted for around 13.73% of all terminal recorded data.

PCA was then applied to the terminal dataset to reduce the dimensions and identify the lead variables of the terminal data. Using 58% data variability, five principal components (PCs) were discovered and used. The PCs consisted of transmit, receive, and signal-to-noise ratio (SNR) attributes. It should be noted that there were many SNR variables with each measuring an aspect of the network performance.

### 4.3. Pattern Recognition and Prediction Algorithms

As noted in Section 2, there are a variety of machine learning algorithms. However, each has its own weaknesses and strengths. As a result, a variety of classification and regression algorithms were selected for the prediction and pattern recognition algorithms in the proposed architecture.

1. **Classification:** Five classification algorithms were considered and compared: classification decision tree, logistic regression, SVM, naïve Bayes and random forest classification tree. In order to apply classification algorithms, an additional variable called IS_DISRUPTED was added to the data. This variable takes a value of 0 when a terminal status flag (the status flag is generated using a type of error checking algorithm) is equal to 0 (not disrupted), taking a value of 1 when a terminal status flag is set to any positive value different than 0 (disrupted). This approach was used because classification algorithms need labelled data to predict the class into which a data entry might fall. By following this approach, it was possible to use the values of the network variables to classify an entry as either disrupted or not disrupted.

Accuracy (Equation (2)) and precision metrics were tools that were used to evaluate performance. Precision (Equation (6)) indicates the proportion of true positives (Equation (3)) among classified as positive, while recall (Equation (7)), another applied tool, indicates true positives (Equation (3)) among all positive instances. These performance matrices examine different criteria. While the accuracy cannot reflect how well the classifier performed for positive classification, precision and recall can. Table 2 shows that SVM has a quite high accuracy, but very low precision and recall, which means that the method classified all instances as not disrupted. Naïve Bayes has high recall, but low 𝒜 and precision, which indicates large number of false positives. Logistic regression and random forest algorithms have low recall and therefore have numerous false negatives.

(2) $$𝒜=\frac{Number\;of\;correct\;predictions}{Total\;number\;of\;predictions\;made}$$

(3) $$\mathrm{True}\;\mathrm{Positive}\;\mathrm{Rate}=\frac{Number\;of\;True\;Positives}{Number\;of\;False\;Negatives+Number\;of\;True\;Positives}$$

(4) $$\mathrm{True}\;\mathrm{Negative}\;\mathrm{Rate}=\frac{Number\;of\;True\;Negatives}{Number\;of\;True\;Negatives+Number\;of\;False\;Positives}$$

(5) $$\mathrm{False}\;\mathrm{Positive}\;\mathrm{Rate}=\frac{Number\;of\;False\;Positive}{Number\;of\;True\;Negatives+Number\;of\;False\;Positives}$$

(6) $$\mathrm{Precision}=\frac{True\;Positives\;Rate}{True\;Positive\;Rate+False\;Positive\;Rate\;}$$

(7) $$\mathrm{Recall}=\frac{True\;Positives\;Rate}{True\;Positive\;Rate+False\;Negative\;Rate\;}$$

A confusion matrix is shown for all algorithms in Table 3. In this work, the classification decision tree algorithm was considered as a good method for terminal data analysis because it obtains high accuracy, appropriate precision and recall, and good results in confusion matrix, compared to other algorithms, and therefore was considered for further analysis.

**Figure 5:**
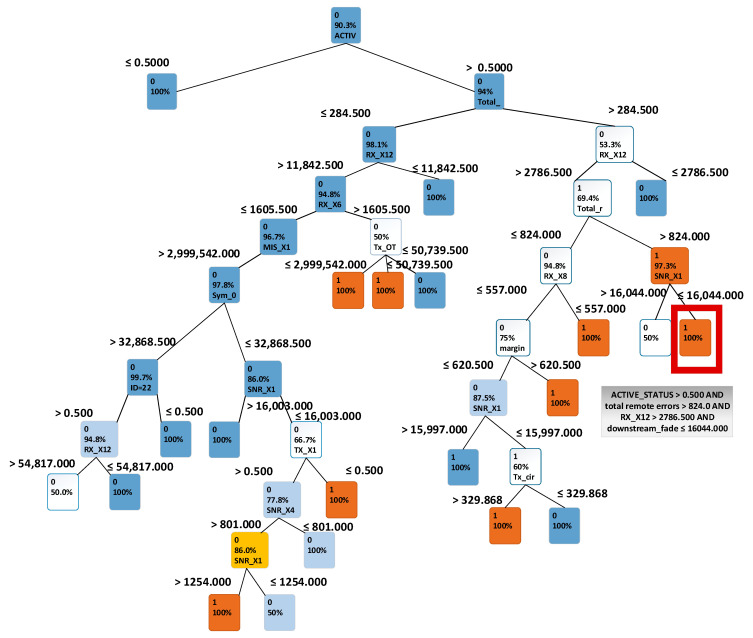
Classification decision tree for terminal data, with IS_DISRUPTED variable as a target.

Results of using the classification decision tree technique are presented in Figure 5. By following the tree, one can see which terminal variables lead to possible disruptions. For example, the highlighted node in red in Figure 5 indicates that if a terminal’s ACTIVE_X1 is greater than 0.5, MIS_X7 (a variable in the dataset indicating equipment error) is greater than 824, RX_X12 (a variable in the dataset indicating received packet) is greater than 2786.5 and FADE_X1 is smaller or equal to 16,044, then the terminal will be disrupted and present a data mismatch greater than 0. This was an interesting finding, because it shows that data mismatches depend on errors in other terminals. This implied that errors might propagate through the network. This finding also supported the experience of the satellite engineers in that each network has its own behavioural pattern.

The other characteristics considered essential in disruption prediction are: RX_X8, TX_X6 (A transmit variable), RX_X18, MIS_X6, MIS_X1, MIS_X8, and SNR_X1 (a signal to noise variable). A disruption is usually observed when the traffic indicators are low (RX_X18, RX_X8, TX_X6) or when downstream signal quality is low (SNR_X1). Because a decision tree provides a transparent view of the decision process, it enabled clearer understanding of why a specific phenomenon occurs and the dependencies between a target variable and other variables.

2. **Regression:** In this work, regression was used to estimate the exact value of a target characteristic which was disrupted (1) and not disrupted (0). RMSE, the root mean squared error, was used to evaluate how close the prediction is to the actual value. The smaller value, the better the prediction. Three regression algorithms, regression tree, linear regression, and SVM regression, were assessed in the experiments.

Using other terminal attributes, regression was used to predict the value of the RX_15 terminal variable. A nonzero value of RX_15 is a sign of serious problems occurring in the network. It was discovered that the regression tree had the best performance among other methods, as it creates the smallest error (Table 4).

### 4.4. Working Memory, Inference Engine, and Visual Notifications

In this section, we discuss how we applied the working memory, inference engine, and notification modules of our architecture to this case study.

After analysing the models generated by different pattern recognition and prediction algorithms, it was discovered that for this case study, models generated by classification decision trees and regression decision trees offered the best results. As a result, these algorithms were used to generate models for the working memory section of the proposed methodology. The generated models were used for analysing aggregated data bundles (ADBs) that were held-out during training. These ADBs served as the testing dataset for the generated models. The ADBs were passed to the generated models in chunks. The generated models were used for identifying network disruptions and transients in the satellite network caused by abnormal behaviour or environmental factors such as passing clouds.

(1) Identifying disruptions: A disruption means a terminal suffers a hardware fault. In this section, how the knowledge of the network and classification decision trees were used to identify faults in a network is discussed. As discussed in the proposed framework section, the functional map of the network was used to derive a simple rule in this work. The rule states that “if a terminal is predicted to belong to another network different from its assigned network, then it is assumed that either the model that did the prediction is wrong or there is something wrong with the terminal”.

**Figure 6:**
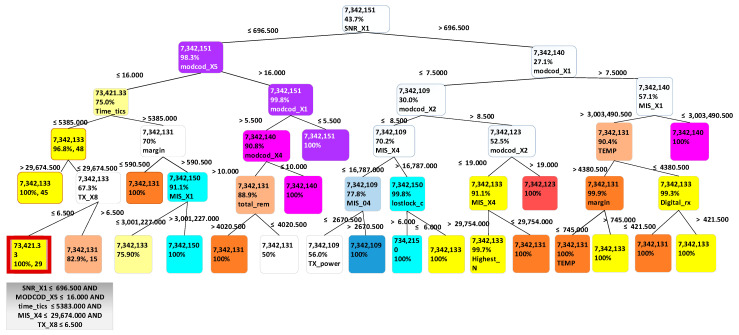
Classification decision tree for all sub route groups for many terminals.

An important advantage of using decision tree is the transparency of the decision process. By using data containing all sub route groups and terminal data, a trained decision tree can be built to enable users to easily observe which sub route group a particular data entry belongs to, and highlights the important characteristics. The decision tree, illustrated in Figure 6, highlights that SNR_X1, MIS_X4, MIS_X1, MODCOD_X4, MODCOD_X5, TEMP, MIS_X5, MIS_X6, TIME_X1, MIS_X7, RX_X16, RX_X12, and TX_X3 are characteristics that distinguish between specific sub route groups.

For example, in Figure 6, when a data entry has SNR_X1 smaller or equal to 696.5, MODCOD_X5 smaller or equal to 16, TIME_X1 smaller or equal than 5385, MIS_X4 smaller or equal to 29674.5, and RX_X17 smaller or equal to 6.5, then this entry will belong to sub route group 7342133. This tree representation enables the highlighting of terminal characteristics to enable users to distinguish between specific sub route groups. It was discovered that for the training dataset used, SNR_X1, MODCOD_X1, and MODCOD_X5 are the main characteristics that can be used in sub route group distinction. This is because they are located at the top of the tree.

Using the decision tree as a predictor, ADBs containing terminal data were classified into specific sub route groups based on characteristics. There were in total 89531 correctly classified entries and 10,469 misclassified entries, which give 0.8953 accuracy for the method. Table 5 presents the confusion matrix representing how the terminals were classified into sub route groups. By using the earlier rule that states “if a terminal is predicted to belong to another network different from its assigned network, then it is assumed that either the model that did the prediction is wrong or there is something wrong with the terminal”, all entries that were misclassified were deemed as a good source of information relating to outliers or problem scenarios. If a terminal was classified to a wrong sub route group it means that some of its characteristics were not typical for that group, thereby indicating a problem.

**Figure 7:**
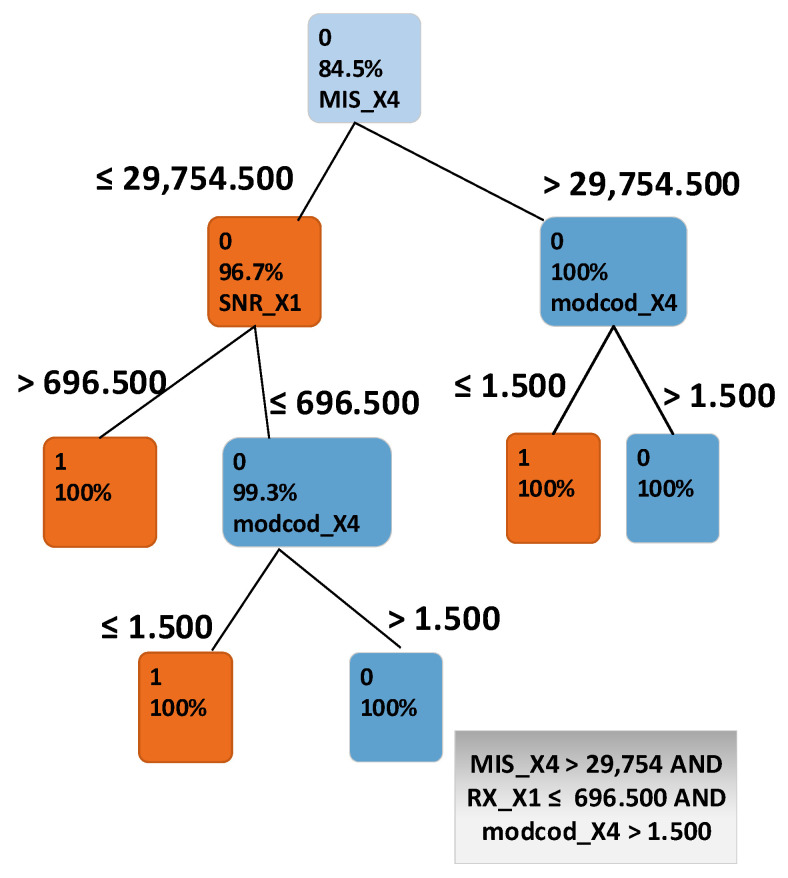
Classification decision tree for instances of sub route group SUB_GRP_2.

Through analysing accurate classifications and misclassifications, we were able to drill into a specific sub route group and discover which attributes are critical in network disruption detection. The analysis was performed using sub route group SUB_GRP_2, as it has the highest number of misclassifications. The classification decision tree in Figure 7 shows that the attributes MIS_X4, SNR_X1 and MODCOD_X4 made a major difference between misclassified instances and those that were classified correctly. Unlike the previous single threshold scheme used by the satellite engineers, this showed that there were a number of attributes that could affect a terminal’s performance.

Using the misclassified terminals, the next step was to distinguish which state the terminal was in—was it experiencing a hardware fault, undergoing short term transient conditions in at the geographical location (e.g., passing rain clouds), or was it experiencing a spatiotemporal condition such as a tree growing in front of its antenna.

(2) Identifying transients: We assumed that the terminals in the network could be in three different states: a normal working state, a transient state with cloud covering or tree waving in front of satellite dish, and an abnormal (broken) state. In this work, SNR_X1 was identified as one of the crucial variables in the dataset. This was determined through PCA and the analysis that was conducted in earlier sections. As a result, SNR_X1 was investigated to find out if this variable could be used to estimate either a short transient or spatiotemporal conditions happening in the vicinity of the terminals, such as cloud covering or a hardware failure caused by a broken terminal. A k-means clustering algorithm was used to derive two clusters, C1 (abnormal) and C2 (normal), from the ADBs. The clustered data were used as input for the classification decision tree, which enabled us to analyse which ranges of SNR_X1 that the majority of the clustered data belonged to.

**Figure 8:**
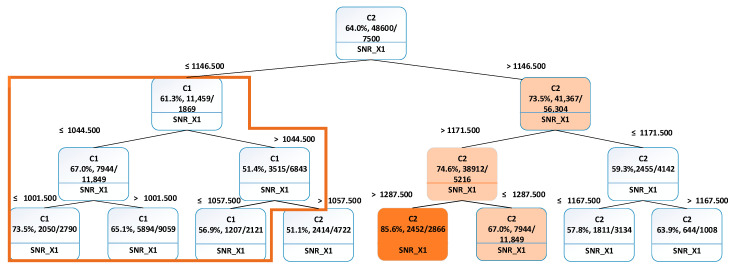
Classification decision tree of two clusters described by SNR_X1.

**Figure 9:**
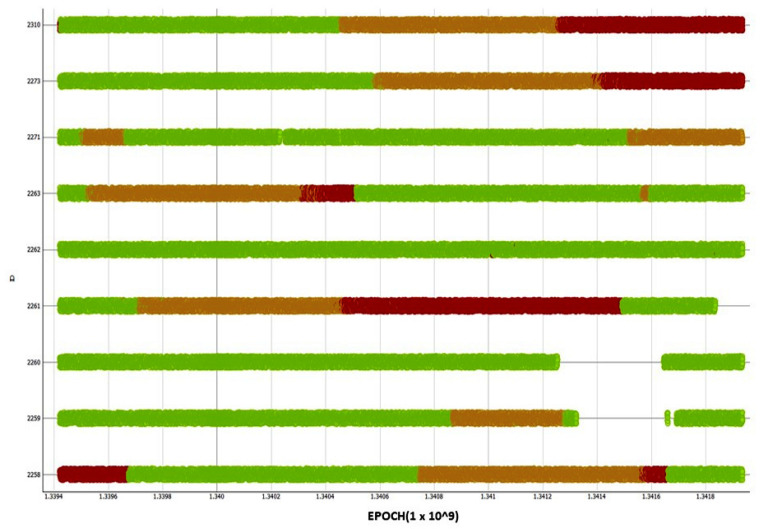
Time series data for various terminal IDs vs. time. Green sections are normal working conditions, amber sections are cloud cover, and red is either disruption or heavy cloud cover affecting signal quality, e.g., 2263.

**Figure 10:**
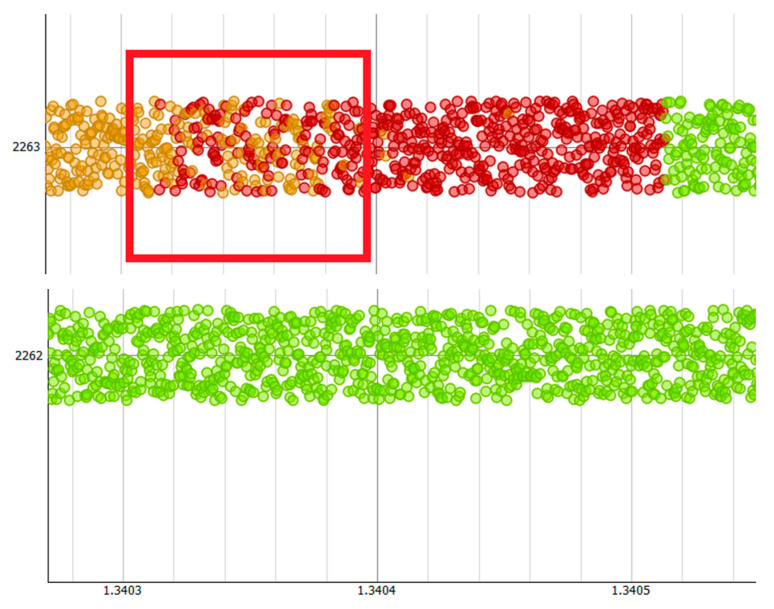
Plot showing how terminal 2263 transitions from amber (cloud cover) to red (heavy cloud cover).

The decision tree is shown in Figure 8. The root of the tree marked in Figure 7 shows which cluster the majority of the data belongs to. In this particular case, it is shown that 64.8% of all data entries belong to cluster C2 (normal). As the majority of entries belong to cluster C2, it is assumed that this cluster represented “normal network behaviour”. The cluster C1 (abnormal) contains remaining 35.2%, which can be considered as an outliers group with possible signal quality issues. K-means method was then applied to derive two clusters- C1a (clouds) and C1b (disruptions) from the C1 cluster. The clustered data from the C1 cluster were used to build a classification decision tree, which classifies which cluster a new terminal’s data set belongs to. The classification decision tree was then used to tag the terminal time series data. Figure 9 presents the time vs Terminal ID, which allows users to observe that the states of all terminals vary as time changes. Some terminals might be in similar locations, as they exhibit similar disruption patterns. For example, terminals 2310 and 2273 were classified as cloud cover state and later they were classified to the disrupted state at similar times. Some terminals exhibit transition phases, when the cloud and disruptions occur simultaneously (Figure 10).

These visual representations and colour-based notifications provided an engineer a high level view of the satellite network from a terminal perspective. As a result of the colour scheme used, the engineer could focus on problematic terminals for further investigation.

## 5. Discussion and Summary

As more cyber–physical systems are created and connected to each other, the amount of data generated will also increase. As these systems become more connected and intertwined, complex systems will emerge. Such complex systems will become increasingly difficult to monitor. This will also lead to an increase in the possibility of cyberattacks [30]. The proposed knowledge-based architecture shown in Figure 3 could provide a paradigm that can be extended to different monitoring systems for different purposes and application domains. It could also be used to augment engineers in identifying anomalies in a large cyber–physical system. With such an autonomous system platform, it is proposed that data scientists can have a foundation to develop and deploy data informed models for various use cases.

Our proposed architecture can be adapted for various purposes and offers a way to integrate expert domain knowledge, via the knowledge base and inference engine, into the results produced by the machine learning algorithms. The rules also provide context for the data and supports a human engineer in understanding the results generated by the automated agent.

The working memory module offers an area in which models of pattern recognition and prediction algorithms can be deployed and validated using real-time data. Due to the nature of this module, multiple or hybrid models can be constructed for different purposes, depending on the use case and the need of users. We used a variety of algorithms such as decision trees, logistic regression, SVM, naïve Bayes and random forest to demonstrate the **flexibility of adding intelligent algorithms to the architecture**. The transparency of decision trees enabled us to understand why a specific phenomenon occurred and the dependencies between a target variable and other variables in the domain. A regression approach enabled users to predict whether a terminal will be disrupted or not. The SVM regression method did not perform well in this case, but it might provide an appropriate approach for other use cases. Furthermore, the use of a working memory enables the testing of various models in order to decide which to carry forward.

**Figure 11:**
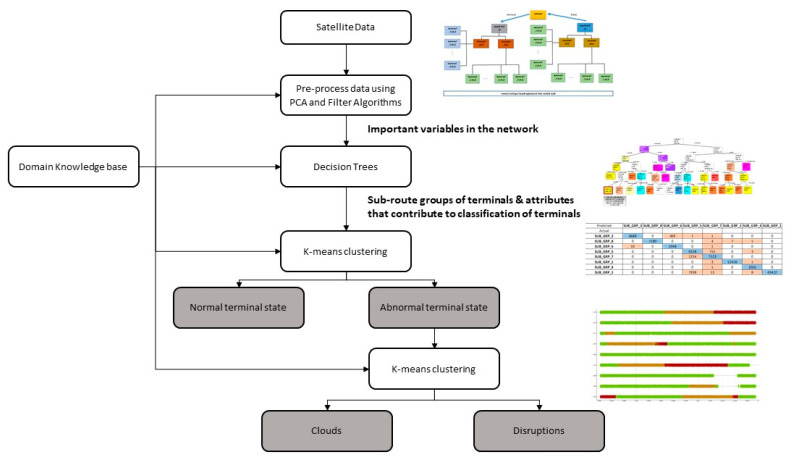
A summary of the process used to identify terminal states in a large satellite network.

By combining working memory, knowledge base, and inference engine, the experimental results reveal that sub route groups and networks are **unique and follow specific patterns and behaviour that can be extracted using classification algorithms**. By making use of this observation, classification models were used in anomaly detection, whereby misclassifications are treated either as outliers or disruptions. Nevertheless, finding the differences between outliers and regular data indicating permanent disruption (hardware faults) or temporary disruption (via intermittent clouds) was key in this work. As a result, use was made of k-means clustering algorithms and subsequently classification algorithms to enable the development of models to identify terminal transition between normal working conditions and clouds or total disruption. By making use of the derived model, it was possible to provide color-coded time series visualisation to reveal what type of disruptions took place at different times. It was revealed that some terminals exhibit transition phases where the cloud cover and disruptions occur simultaneously. This could be during heavy downpours. Such color-coded time series could form part of the notification module to support engineers. As the use of machine learning is heavily dependent on data, the pre-processing module is very important. This module enables the removal of erroneous values and a reduction in the dimension space of the large cyber–physical system involved in this research. This is perhaps one of the most important modules in the architecture as without it, the algorithms in the pattern recognition and prediction modules will be ineffective. The results from this study were confirmed by the satellite engineers through the use of a single blind experimental setup. Figure 11 shows a summary of the steps that were followed. This approach could be applied to other systems in which abnormal behaviours need to be recognised.

As would be seen, domain knowledge is necessary throughout the process. Furthermore, it should be noted that because the approaches we used in this paper were data-driven, the models could potentially be updated continuously. **This creates a system that learns continuously and that able to self-adapt based on new data from the network**. The machine learning approaches we have used in this work, particularly the decision tree, offers an engineer ability to sense-check that the generated classification models are correct. In this situation, the engineer would use his experience of the satellite network to ensure that the attributes used by the model makes sense. **In other words, this offers readability and interpretability to the engineer**. As stated in the case study section, it was discovered that unlike the single variable that the engineers were using, there were a multiple of variables that contributed to the state of terminals in the network. The visualisation and notification approach provided in Figure 9 also offers a condensed view of the network to the engineer. It highlights the states of the terminals in the network and any potential issues. These results show the feasibility of using an autonomous system to differentiate a normal state, cloud cover, and disruption.

In summary, our architecture offers (1) flexibility to add new or extend existing machine learning algorithms to meet the user needs, (2) an enhanced pattern recognition and prediction through the support of machine learning algorithms and the expert knowledge on satellite infrastructure, (3) the ability to adapt to changing conditions of the satellite network, and (4) the ability to augment satellite engineers through interpretable results.

## 6. Conclusions

In this paper, a knowledge-based cognitive architecture for monitoring a satellite network containing a large number of terminals is developed. The large number of terminals is such that it is not possible to monitor by human means alone. An important advantage of the proposed architecture is that it allows humans to be involved in the final decision under the combination of the decision generated by machine learning models and knowledge-based rules. The function of the architecture is not to replace the human engineers monitoring the network, but to augment their capability in monitoring such a large network. It is demonstrated how the various concepts of the proposed architecture can be utilised to develop capabilities for anomaly detection through a case study. The proposed architecture can be a paradigm for monitoring various IoT enabled cyber–physical systems in different application domains and thus meet the requirement of trustworthiness of increasingly complex cyber–physical systems. The proposed system could also help to overcome the challenge of monitoring and sense-making in an environment of exponential data generation rates. Furthermore, in order to aid interpretability, the structure of the network was taken into consideration during the construction of the knowledge-based cognitive architecture. The advantage of this approach was that it enabled the engineer to further understand the results produced by the algorithms operating in the cognitive architecture. It was shown in literature that embedding knowledge of the domain into cognitive architectures could further improve interpretability [31].

## Figures and Tables

**Table 1:** Showing sub route groups, networks, and number of terminals.

SUB_ROUTE_GROUPS	Network	Number of Terminals
SUB_GRP_1	NET_1	36
SUB_GRP_2	NET_2	432
SUB_GRP_3	NET_3	7
SUB_GRP_4	NET_4	1
SUB_GRP_5	NET_5	15
SUB_GRP_6	NET_6	4
SUB_GRP_7	NET_7	13
SUB_GRP_8	NET_8	13

**Table 2:** Performance of classification algorithms.

Algorithm	𝒜	Precision	Recall
Classification Decision Tree	0.998	0.838	0.814
Logistic Regression	0.996	0.829	0.486
SVM	0.993	0.000	0.000
Naïve Bayes	0.685	0.019	0.871
Random Forest	0.997	1.000	0.543

**Table 3:** Confusion matrix for classification algorithms.

Algorithm		Predicted	0	1
	Actual			
Classification Decision Tree	0		9918	11
	1		13	57
Logistic Regression	0		9922	7
	1		36	34
SVM	0		9929	0
	1		70	0
Naïve Bayes	0		6789	3140
	1		9	61
Random Forest	0		9929	0
	1		32	38

**Table 4:** Performance of regression algorithms for predicting terminal disruption based on the terminal attribute RX_15.

Algorithm	RMSE
Regression Tree	47.876
Linear Regression	49.372
SVM Regression	154.466

**Table 5:** Confusion matrix in sub route groups.

Predicted	SUB_GRP_3	SUB_GRP_8	SUB_GRP_6	SUB_GRP_5	SUB_GRP_7	SUB_GRP_1	SUB_GRP_4	SUB_GRP_2
**Actual**								
**SUB_GRP_3**	6688	0	469	1	1	0	0	0
**SUB_GRP_8**	0	7180	0	0	4	7	1	0
**SUB_GRP_6**	10	0	2048	0	1	0	0	0
**SUB_GRP_5**	0	0	0	9228	755	0	3	0
**SUB_GRP_7**	0	0	0	1254	7523	0	0	0
**SUB_GRP_1**	0	0	0	0	3	12,416	1	0
**SUB_GRP_4**	0	0	0	0	1	0	1026	0
**SUB_GRP_2**	0	0	0	7938	13	0	8	43,422

## Data Availability

Not applicable.

## References

[1] (2019). SpaceX Mission. https://www.spacex.com/launches/.

[2] (2020). IMO Profile. https://www.imo.org/en/OurWork/Environment/Pages/Default.aspx.

[3] Lázaro F., Raulefs R., Wang W., Clazzer F., Plass S. (2019). VHF Data Exchange System (VDES): An enabling technology for maritime communications. CEAS Space J..

[4] (2016). NSR Maritime SATCOM Markets. https://www.nsr.com/research_cat/satellite-communications-reports/.

[5] (2019). NSR Maritime SATCOM Markets, 7th ed. https://www.nsr.com/research_cat/satellite-communications-reports/.

[6] Sotelo Monge M.A., Maestre Vidal J., García Villalba L.J. (2017). Reasoning and knowledge acquisition framework for 5G network analytics. Sensors.

[7] Fu T.C. (2011). A review on time series data mining. Eng. Appl. Artif. Intell..

[8] Batal I., Fradkin D., Harrison J., Moerchen F., Hauskrecht M. Mining recent temporal patterns for event detection in multivariate time series data. Proceedings of the 18th ACM SIGKDD International Conference on Knowledge Discovery and Data Mining.

[9] Abdi H., Williams L.J. (2010). Principal component analysis. Wiley Interdiscip. Rev. Comput. Stat..

[10] Kudo T., Morita T., Matsuda T., Takine T. PCA-based robust anomaly detection using periodic traffic behavior. Proceedings of the IEEE International Conference on Communications Workshops (ICC).

[11] Bagane S.A., Patil S. (2015). Detection of Anomalies using Online Oversampling PCA. Int. J. Sci. Eng. Technol. Res. (IJSETR).

[12] Munz G., Li S., Carle G. Traffic Anomaly Detection Using K-Means Clustering. Proceedings of the GI/ITG Workshop MMBnet.

[13] Syarif I., Prugel-Bennett A., Wills G. (2012). Data mining approaches for network intrusion detection from dimensionality reduction to misuse and anomaly detection. J. Inf. Technol. Rev..

[14] Habeeb R.A.A., Nasaruddin F.H., Gani A., Amanullah M.A. (2019). Clustering-based real-time anomaly detection—A breakthrough in big data Technologies. Trans. Emerg. Telecommun. Technol..

[15] Liu X., Nielsen P.S. (2016). Regression-based Online Anomaly Detection for Smart Grid Data. arXiv.

[16] Kromanis R., Kripakaran P. (2013). Support vector regression for anomaly detection from measurement histories. Adv. Eng. Inform..

[17] Khoshgoftaar T.M., Allen E.B., Deng J. (2002). Using regression trees to classify fault-prone software modules. IEEE Trans. Reliab..

[18] Bergman L., Hoshen Y. (2020). Classification-based Anomaly Detection for General Data. arXiv.

[19] Roberts E., Bassett B.A., Lochner M. (2019). Bayesian Anomaly Detection and Classification. arXiv.

[20] Al Mamuna S.M.A., Valimaki J. Anomaly Detection and Classification in Cellular Networks Using Automatic Labeling Technique for Applying Supervised Learning. Proceedings of the Complex Adaptive Systems Conference with Theme: Cyber Physical Systems and Deep Learning, CAS2018.

[21] Capozzoli A., Lauro F., Khan I. (2015). Fault detection analysis using data mining techniques for a cluster of smart office buildings. Expert Syst. Appl..

[22] Samantaray S.R. (2009). Decision tree-based fault zone identification and fault classification in flexible AC transmissions-based transmission line. IET Gener. Transm. Distrib..

[23] Moeyersoms J., de Fortuny E.J., Dejaeger K., Baesens B., Martens D. (2015). Comprehensible software fault and effort prediction: A data mining approach. J. Syst. Softw..

[24] Feng W., Zhang Q., Hu G., Huang J.X. (2014). Mining network data for intrusion detection through combining SVMs with ant colony networks. Future Gen. Comput. Syst..

[25] Purarjomandlangrudi A., Ghapanchi A.H., Esmalifalak M. (2014). A data mining approach for fault diagnosis: An application of anomaly detection algorithm. Measurement.

[26] He H., Tiwari A., Mehnen J., Watson T., Maple C., Jin Y., Gabrys B. Incremental Information Gain Analysis of Input Attribute Impact on RBF-Kernel SVM Spam Detection. Proceedings of the 2016 IEEE Congress on Evolutionary Computation (CEC).

[27] Gaber M., Zaslavsky M.A., Krishnaswamy S. (2005). Mining data streams: A review. ACM Sigmod Rec..

[28] Weiss G.M. (2005). Data mining in telecommunications. Data Mining and Knowledge Discovery Handbook.

[29] Kalegele K., Sasai K., Takahashi H., Kitagata G., Kinoshita T. (2015). Four decades of data mining in network and systems management. IEEE Trans. Knowl. Data Eng..

[30] Holmes M. (2020). The Growing Risk of a Major Satellite Cyber Attack, Via Satellite. http://interactive.satellitetoday.com/the-growing-risk-of-a-major-satellite-cyber-attack/.

[31] Chao M.A., Kulkarni C., Goebel K., Fink O. (2019). Hybrid deep fault detection and isolation: Combining deep neural networks and system performance models. arXiv.

